# Influential and theoretical analysis of nano-defect in the stub resonator

**DOI:** 10.1038/srep30877

**Published:** 2016-08-01

**Authors:** Hui Xu, Hongjian Li, Boxun Li, Zhihui He, Zhiquan Chen, Mingfei Zheng

**Affiliations:** 1 College of Physics and Electronics, Central South University, Changsha 410083, PR China

## Abstract

We investigate a classic optical effect based on plasmon induced transparency (PIT) in a metal-insulator-metal (MIM) bus waveguide coupled with a single defective cavity. With the coupled mode theory (CMT), a theoretical model, for the single defective cavity, is established to study spectral features in the plasmonic waveguide. We can achieve a required description for the phenomenon, and the theoretical results also agree well with the finite-difference time-domain (FDTD) method. Our researches show that the defect’s position and size play important roles in the PIT phenomenon. By adjusting the position and size of the defect, we can realize the PIT phenomenon well and get the required slow light effect. The proposed model and findings may provide guidance for fundamental research of the control of light in highly integrated optical circuits.

Electromagnetically induced transparency (EIT) occurs in atomic systems is attributed to the quantum interference between the excitation pathways to the atomic upper levels^1^,^2^. Due to the interesting physics and important applications, the EIT effect has received much attention. But the harsh experimental conditions demanded to observe the EIT effect baffle its practical application, which leads to a rapid progressive research for theoretical analysis on EIT^3^,^4^. It is worth noting that controlling light in nanoscale structure is very necessary for highly integrated optics. Due to the strong dispersion and slow-light propagation within the transparency window, EIT promises a variety of potential applications in optical data storage, ultrafast switching, and nonlinear optical processes^5^,^6^. In recent years, theoretical analysis and experimental results show that a novel phenomenon analogous to EIT can also occur in dielectric photonic resonator systems, which is known as the plasmon induced transparency (PIT)^7^,^8^,^9^,^10^.

Surface plasmon polaritons (SPPs) are waves trapped on the surfaces of metals owing to the interaction between the free electrons in metal and electromagnetic field in dielectric^11^,^12^. In another word, SPPs are always involved in the metal-based devices and known as surface electromagnetic waves which are well confined to metal-insulator interfaces. Due to the capability of confining light beyond the diffraction limit and manipulation of light in nanoscale domain, SPPs can be regarded as a promising candidate for integrated plasmonic device^13^,^14^. Among the different plasmonic devices, Metal-Insulator-Metal (MIM) waveguides have attracted considerable attention, because they support modes with deep wavelength scale and an acceptable length for SPPs propagation. In view of the unique features of MIM waveguides, the PIT observed in nanoscale plasmonic resonator systems has been theoretically predicted and experimentally illustrated in recent researches^15^,^16^,^17^,^18^,^19^,^20^. Cao *et al*. analyzed plasmon-induced transparency in a single multimode stub resonator^18^. Lu *et al*. demonstrated plasmonic analog of electromagnetically induced transparency in multi-nanoresonator-coupled waveguide systems^19^. Zhan *et al*. achieved sensing analysis based on plasmon induced transparency in nanocavity-coupled waveguide combined with the transfer matrix method^20^. He *et al*. attested theoretical analysis for plasmon-induced transparency in waveguide systems^8^. However, very few qualitative descriptions have been performed on the function of direct coupling in plasmonic waveguide with a single defective cavity.

In this paper, we investigate the PIT transmission and slow-light effect in the plasmonic resonator system, which consists of a MIM bus waveguide coupled with a single defective cavity. In order to facilitate the theoretical calculation and numerical simulation, we simplify the defects into a regular block. The CMT results show that the formation of transparency window is attributed to the defect’s position and the evolution of transparency is mainly determined by the variation of defect’s size. To confirm the correctness of the theoretical description, we have compared them with the FDTD simulations^21^. Finally, with the group index in this coupling scheme, we also discuss slow-light effect of the scheme.

## Structure and Theory Model

**Figure 1:**
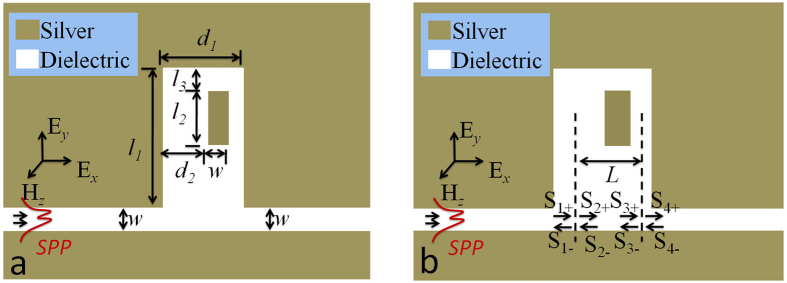
(**a**) Schematic of MIM waveguide coupled to a single defective cavity. Figure 1b Equivalent theoretical model for Figure 1a.

Figure 1a is the schematic illustration of our system. It consists of a MIM bus waveguide with a single defective cavity. The metal and insulator are selected as silver and air, respectively. The frequency dependent permittivity of the silver is approximated by the Drude model^7^,^8^,^9^, which defines as *ε*(*ω*) = *ε*_∞_ − *ω*_*p*_^*2*^*/*(*ω*^*2*^ + *iωγ*_*p*_), where *ω* stands for the angle frequency of the incident wave, the dielectric constant at the infinite frequency *ε*_∞_ = 3.7, the plasma frequency *ω*_*p*_ = 1.38 × 10^16^ rad/s, and the damping rate *γ*_*p*_ = 2.73 × 10^13^ rad/s. The width *w* for both the bus waveguide and the defect is 50 nm. The length of the cavity are selected as *l*_*1*_ = 425 nm. And these values are constant throughout the full text. The other structure parameters are the width of the cavity (*d*_1_), the distance between the cavity’s left edge and defect (*d*_*2*_), length of the defect (*l*_*2*_), and the distance between cavity’s upper edge and defect (*l*_*3*_). When the TM-polarized wave is injected along x-axis, SPP waves can be formed on the metal-insulator interfaces and propagate confined in the waveguide.

As the SPP waves pass through the bus waveguide, the energy can be coupled into the cavity and the dynamic transmission characteristics of the proposed structure can be investigated by the CMT^22^,^23^. As shown in Fig. 1b, the incoming and outgoing waves in the resonators are depicted by S_*j*±_ (*j* = 1, 2, 3, 4). The subscript ± represent two propagating directions of waveguide modes, as shown in Fig. 1. Thus, the energy amplitude *a*_*n*_of the *n*th resonator (*n* *=* 1, 2) can be expressed as


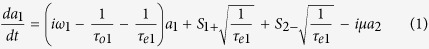



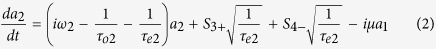


here, *ω* is the angular frequency of the input optical pulse, *ω*_*n*_ (*n* = 1, 2) is the nth resonant angular frequency, 1/*τ*_*on*_ = *ω*_*n*_/(2*Q*_*on*_) and 1/*τ*_*en*_ = *ω*_*n*_/(2*Q*_*en*_) (*n* = 1, 2) are the internal loss and the coupling loss, respectively. *Q*_*on*_, *Q*_*en*_ are the related quality factors. *μ* is the coupling coefficient between the two resonant modes. With the conservation of energy, they also satisfy the following relations


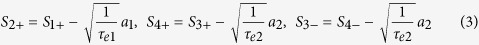



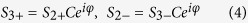


where *φ* = Re(*β*_*spp*_)*L* = *ω*Re(*n*_*eff*_)*L*/*c* represents the phase shift, C is the related attenuation coefficient, *L* is the coupling distance between the two resonant modes and *c* is the light velocity in vacuum. *β*_*spp*_ and *n*_*eff*_ are propagation constant and effective index for SPPs^24^,^25^, respectively.

From equation (1, 2, 3, 4) and the condition that the light is only injected from the left port (*S*_*4−*_ = 0), we can achieve the transfer function of this system:


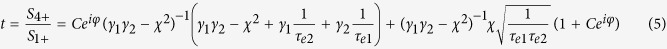


where 
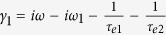
, 
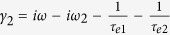
, and 
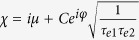
. Thus, the transmission coefficient


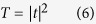


## Results and Analysis

**Figure 2:**
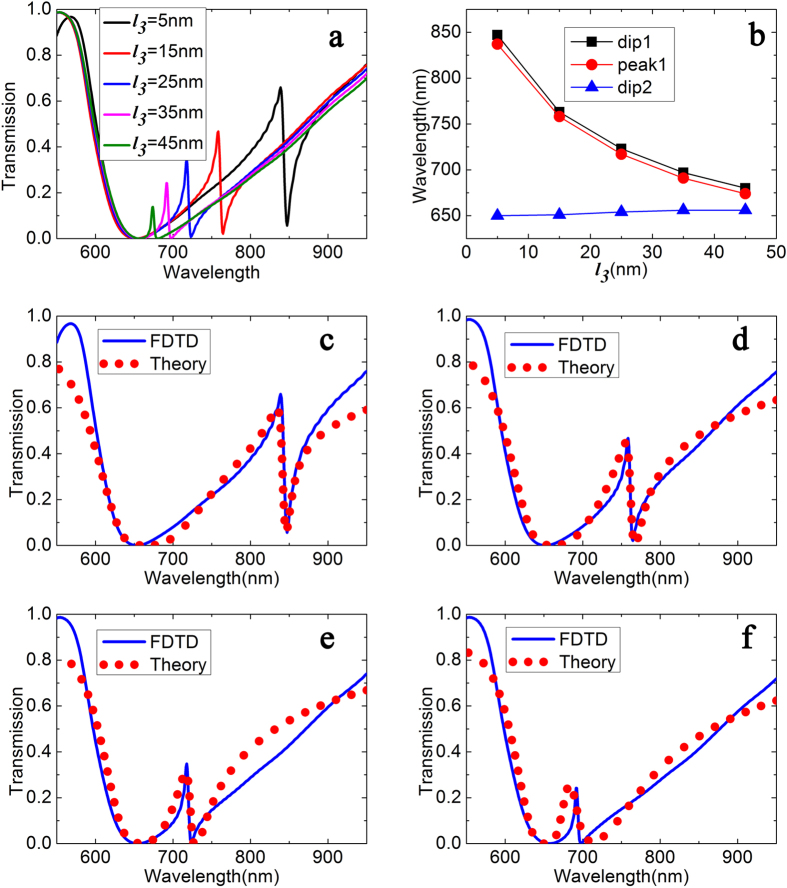
(**a**) Transmission spectra with different length *l*_3_ (5, 15, 25, 35, 45 nm) in defect-resonator-coupled waveguide (*d*_2_ = 110 nm, *l*_2_ = 100 nm). (**b**) The wavelength values of dip and peak as a function of length *l*_*3*_. (**c**–**f**) represent the simulated transmission (blue solid lines) and theoretical fitting (red cycles lines) as *l*_*3*_ = 5 nm, 15 nm, 25 nm, 35 nm, respectively.

Based on the FDTD method, we show the transmission spectra in Fig. 2a. The length *l*_*3*_ varies from 5 nm to 55 nm, the coupling distance is considered as L = 50 nm, and the width of the cavity are selected as *d*_1_ = 200 nm. The wavelength values of dip and peak as a function of length *l*_*3*_ are plotted in the Fig. 2b. In Fig. 2c–f, the blue solid line and red circles lines are calculated by the FDTD and the CMT method, respectively, and we can see the theoretical results are in good agreement with the FDTD simulations. We can also find that the transmission spectra exhibit PIT shapes, namely, a transparency peak in the center of a transmission dip. It is worth noting that a short length induces a broader spectra bandwidth and an increasing peak transmission.

To get more insight into the physics of the observed PIT transmission, the wavelength of transmission dips and peaks as a function of length are plotted in Fig. 2b. In order to distinguish, the dip which has a smaller full width at half maximum is named as dip1 and the other one is named as dip2. The peak which is located between dip1 and dip2 is called as peak1, the following definition of dip and peak is also like this. In Fig. 2b, the black, red and blue lines correspond to the resonance wavelengths of dip 1, peak 1 and dip 2, respectively. When the length *l*_*3*_ varies from 5 nm to 45 nm, dip1 are located in 847 nm, 763 nm, 723 nm, 697 nm, 680 nm, and dip2 are located in 650 nm, 651 nm, 654 nm, 656 nm, 656 nm, and peak1 are located in 837 nm, 758 nm, 717 nm, 691 nm, 674 nm. It is found that the transmission dip 2 approximately unchanged for its invariable defect’s length, while the transmission dip 1 continually shift towards shorter wavelength for its increasing the length *l*_*3*_. Moreover, the transparent resonance peak 1 exhibits a similar change tendency as dip1. As *l*_*3*_ decreases from 45 to 5  nm, the transparent window presents and becomes increasingly clear, which is due to the increment of coupling coefficient between the two resonance modes, and it is in keeping with the CMT.

**Figure 3:**
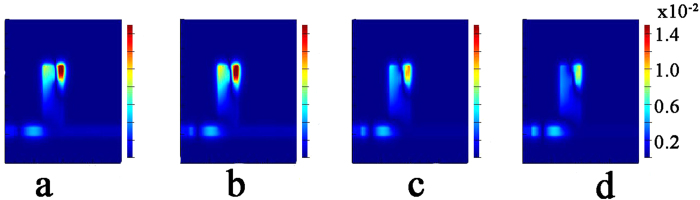
Hz fields **|**H_z_**|** for the defect length of l3 (**a**) 5 nm at the resonant wavelengths of 847 nm, (**b**) 15 nm at the resonant wavelengths of 763 nm, (**c**) 25 nm at the resonant wavelengths of 723 nm, (**d**) 35 nm at the resonant wavelengths of 697 nm.

For the sake of explaining this phenomenon, the H_z_ fields |H_z_| (Here, H_z_ represents the magnetic field at the Z-axis.) at the dip1 are simulated and shown in Fig. 3. From Fig. 3, we can see evidently a standing wave pattern with relative intensities is excited in the rectangular nanocavity because of the structural breaking^26^. In the dip1, most of the energy is located at the narrow between cavity and defect. This research verifies the correctness of the CMT model. What is more, the localized energy becomes stronger along with shorting the value of *l*_*3*_. We can conclude that the defect is the cause of dip1 formation.

**Figure 4:**
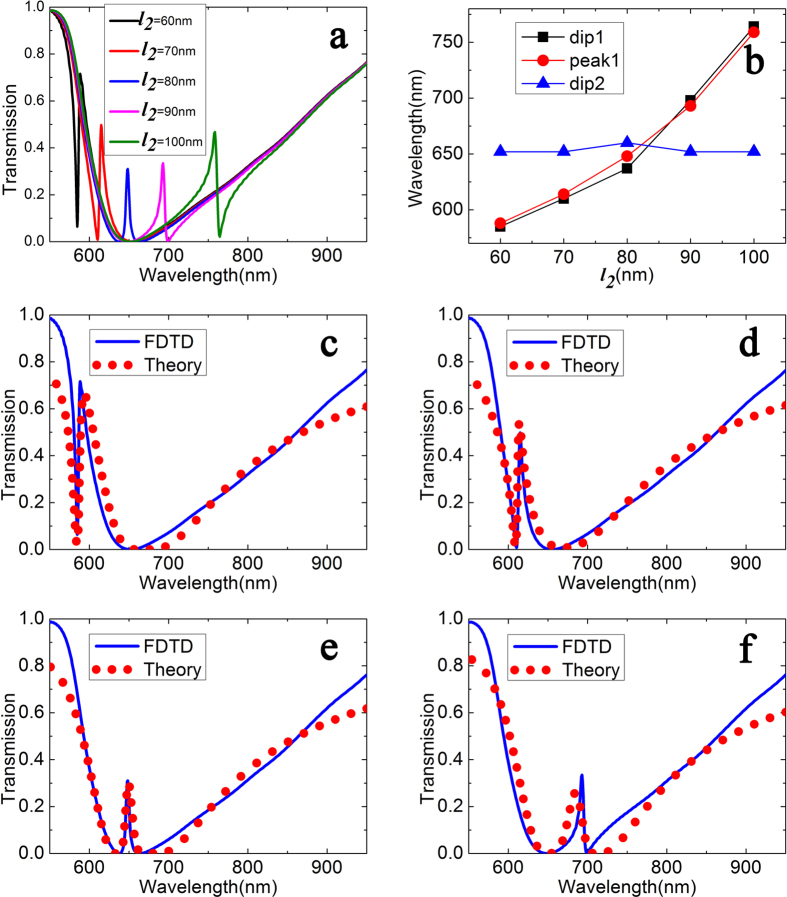
(**a**) Transmission spectra with different defect’s length *l*_*2*_. (**b**) The wavelength values of dip and peak as a function of defect length *l*_*2*_. (**c**–**f**) Simulated transmission (blue solid lines) and theoretical fitting (red circles lines) as *l*_*2*_ = 60 nm, 70 nm, 80 nm, 90 nm, respectively.

In order to get more details to the structure, we change the size of the defect. We are mainly to change the length of the defect, and *l*_*4*_ (*l*_*4*_ = *l*_*1*_ − *l*_*3*_ − *l*_*2*_) is a constant with value as 300 nm. The results are showed in Fig. 4. We can see that there is a quite relationship between the defect’s length and dip 1. As Fig. 4c–f showed, the theoretical results also are in good accordance with the FDTD simulations.

In contrast with the above research, one of dips appears at the shorter wavelength, which is markedly different. From the FDTD simulations and CMT method, we can conclude that defect’s length influences the form of transparency window.

**Figure 5:**
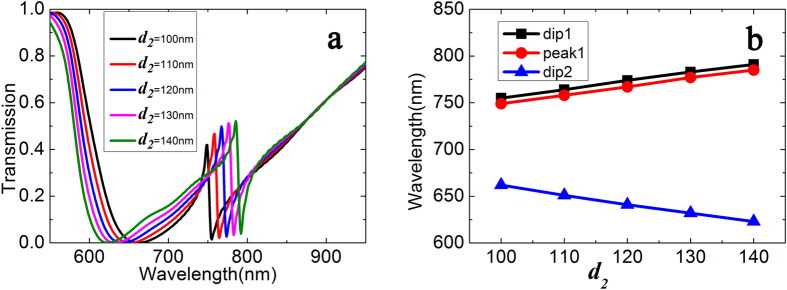
(**a**) Transmission spectra with different width *d*_*2*_ of the cavity. (**b**) The wavelength values of dip and peak as a function of cavity width *d*_*2*_. The black, red and blue lines represent dip1, peak1 and dip2, respectively.

Also, the above studies are based on different characteristics of the defect, and we think what phenomenon would be found if we change size of the cavity. For this reason, we change the width of cavity, and the transmission spectra with different width of cavity are plotted in Fig. 5a. The width of cavity varies 100 nm from 140 nm with a step of 10 nm. From Fig. 5b, it is found that the dip 2 is no longer a constant. The specific numerical values of dip2 are 662 nm, 651 nm, 641 nm, 632 nm, 623 nm. We catch that there is an approximate 10 nm decrease with each increase of the width, but dip1 and peak1 have opposite trends whose increases are approximate 10 nm with the increase of the width.

Slow-light effect is one of the most important applications for PIT in atom system, and our plasmonic system also supports slow group velocities^27^,^28^,^29^,^30^,^31^. The slow- light effect can be described by the group index *n*_*g*_, which can be calculated as


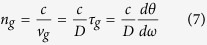


where *ν*_*g*_ is the group velocity, *D* is the length of the plasmonic system, *τ*_*g*_ is the optical delay time, and *θ* stands for the transmission phase shift.

**Figure 6:**
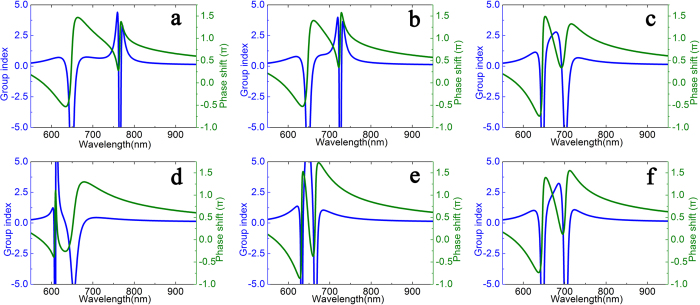
Group index and phase shift (unit of π) in the plasmonic waveguide with a single defective cavity. (**a–c**) correspond to Fig. 2c–e, respectively. And (**d–f**) correspond to and Fig. 3c–e, respectively.

Figure 6 illustrates the group index and the phase shift (units of π) in transparency window. In Fig. 6a–f, we plot the group index as the length between defect and cavity’s upper edge *l*_*3*_ arranges from 15 to 35 nm with 10 nm as the step size and the defect’s length *l*_*2*_ varies from 70 to 90 nm with a step of 10 nm, respectively. And Fig. 6a–c are corresponding to Fig. 2c–e, respectively; Fig. 6d–f are corresponding to Fig. 4c–e, respectively. The corresponding parameters of structure are identical. The other geometrical parameters for the slow-light effect is investigated in our plasmonic system with *d*_*2*_ = 110 nm and *D* = 1100 nm. Obviously, the typical feature of PIT shape is presented, which results from the strong dispersion in the transparency window and the group index can be obtained.

From Eq. (4), we can get the group index *n*_*g*_, which is proportional to the dispersion velocity of transmission phase shift. Figure 6(a–c) depict the group index and the transmission phase shift (*θ* = *arg (t)*) corresponding to Fig. 2(c–e), respectively. And Fig. 6(d–f) represents the group index and the transmission phase shift corresponding to Fig. 4(c–e), respectively. It is clear that the group index is greatly decreased around the PIT dips due to the fast phase changing at the resonances. In Fig. 6a–f, there is a sudden phase jump around the transmission dip, but the normal dispersion in the other wavelength is not obvious. Therefore, one could change the size or position of the defect to realize PIT phenomena, and hence achieve the required slow-light effect. Due to the metal loss included in this MIM system, a negative group index was obtained. It is known as superluminal wave propagation^31^, a common characteristic to lossy metamaterials^32^, and was experimentally observed in atomic EIT systems^33^.

## Conclusion

In summary, the PIT transmission has been numerically and theoretically investigated in the plasmonic system composed of a MIM bus waveguide with a single defective cavity. The CMT and FDTD are utilized in our simulations. For the coupling case, the numerical and theoretical results demonstrate that the formation and evolution of transparency window is attributed to the defect’s position and size. These results may be applied for designing optical switching, optical filter and slow-light devices in highly integrated optical circuits.

## Methods

The frequency dependent optical property of the silver nanostructure is approximated by the Drude model, which defines as *ε*(*ω*) = *ε*_∞_ − *ω*_*p*_^*2*^/(*ω*^*2*^ + *iωγ*_*p*_), where *ω* stands for the angle frequency of the incident wave, the dielectric constant at the infinite frequency *ε*_∞_ = 3.7, the plasma frequency *ω*_*p*_ = 1.38 × 10^16^ rad/s, and the damping rate *γ*_*p*_ = 2.73 × 10^13^ rad/s, which characterizes the absorption loss. The Gauss light source is set at the entrance of the bus waveguide, and a normalized receiving screen is placed at the exit of the bus waveguide. As the input light is incident along x-axis, surface plasmon polaritons can be formed on metal-insulator interface and confined in the waveguide. The characteristic spectra of the structures are found by using the two-dimensional FDTD method with mesh grid size Δx = Δy = 5 nm and Δt = Δx/2c (c is the velocity of light in vacuum). The z-axis can be considered as infinite. The calculated domain is surrounded by perfectly matched layer absorbing boundary. We choose Meep as our FDTD simulation software developed by MIT. And the simulation parameters have been given in our paper.

## Additional Information

**How to cite this article**: Xu, H. *et al*. Influential and theoretical analysis of nano-defect in the stub resonator. *Sci. Rep.* **6**, 30877; doi: 10.1038/srep30877 (2016).
